# Fault Knowledge Graph Construction and Platform Development for Aircraft PHM

**DOI:** 10.3390/s24010231

**Published:** 2023-12-30

**Authors:** Xiangzhen Meng, Bo Jing, Shenglong Wang, Jinxin Pan, Yifeng Huang, Xiaoxuan Jiao

**Affiliations:** Aviation Engineering School, Air Force Engineering University, Xi’an 710038, China; mengxz_sensor@outlook.com (X.M.);

**Keywords:** PHM, knowledge graph, joint extraction of entity relationships, Q&A system

## Abstract

To tackle the problems of over-reliance on traditional experience, poor troubleshooting robustness, and slow response by maintenance personnel to changes in faults in the current aircraft health management field, this paper proposes the use of a knowledge graph. The knowledge graph represents troubleshooting in a new way. The aim of the knowledge graph is to improve the correlation between fault data by representing experience. The data source for this study consists of the flight control system manual and typical fault cases of a specific aircraft type. A knowledge graph construction approach is proposed to construct a fault knowledge graph for aircraft health management. Firstly, the data are classified using the ERNIE model-based method. Then, a joint entity relationship extraction model based on ERNIE-BiLSTM-CRF-TreeBiLSTM is introduced to improve entity relationship extraction accuracy and reduce the semantic complexity of the text from a linguistic perspective. Additionally, a knowledge graph platform for aircraft health management is developed. The platform includes modules for text classification, knowledge extraction, knowledge auditing, a Q&A system, and graph visualization. These modules improve the management of aircraft health data and provide a foundation for rapid knowledge graph construction and knowledge graph-based fault diagnosis.

## 1. Introduction

The demand for highly advanced and high-performance equipment, such as aircraft and weaponry, is increasing due to the rapid advancement of information technology and the implementation of Germany’s ‘Industry 4.0’ and China’s ‘Made in China 2025’ strategies. This demand is particularly relevant in all-airspace, all-weather, multi-element war scenarios. As a result, the system structure of this equipment is becoming increasingly complex. The complexity of the system structure is accompanied by a higher degree of functional integration operating in harsh environments. Consequently, a wide range of failure modes can occur in aircraft and other weaponry, leading to complex fault propagation patterns [1]. These complex and diverse faults pose challenges to the safe and stable operation of the entire aircraft. Efficiently and accurately identifying aircraft faults and enhancing the effectiveness and efficiency of aircraft maintenance is crucial for ensuring stable aircraft operation.

Based on the research into the current state of aircraft field maintenance and guarantee, the current approach still relies on the passive method of “fault-locate the fault-troubleshoot and repair”. When a failure occurs, it necessitates the involvement of a professional maintenance team. In case of a fault, maintenance personnel need to analyze and troubleshoot the fault phenomenon by incorporating relevant historical fault reports and other data. Unfortunately, there remains a dearth of effective intelligent fault diagnosis techniques. Furthermore, the varying proficiency levels of maintenance personnel have resulted in suboptimal stability of the troubleshooting effect. This has also caused maintenance personnel to respond slowly to changes in faults, thereby impeding their ability to promptly and accurately deal with issues [2]. Additionally, given the complex structure of aircraft systems and the varying nature of failures across different systems, aircraft Prognostics and Health Management (PHM) demands a high level of professional expertise from technicians, making it more challenging. Fault diagnosis techniques have evolved from manual testing and external testing to manual diagnosis and intelligent fault diagnosis. Currently, research in the field of Prognostics and Health Management (PHM) primarily focuses on methods based on failure physical models [3] and data-driven approaches [4]. Particularly, migration learning and deep learning methods are utilized to enhance the generalization capability of diagnostic models [5,6,7]. However, physical model-based approaches possess certain limitations. Firstly, they require researchers to possess a comprehensive understanding of the physical structure of the system and the failure mechanism. Secondly, they need to consider various stress factors in the degradation and failure process of the product, in addition to the limited generalization ability of the model. On the other hand, the data-driven approach faces challenges. Due to space and load restrictions on aircraft, it is impractical to install a large number of sensors, making it difficult to obtain sufficient and effective state data for characterizing failures. The existing aircraft systems have accumulated a substantial number of unstructured and semi-structured text-based fault handling cases. Thus, the pressing need in the aircraft PHM field is to swiftly and accurately extract valuable information from this vast amount of text data [8,9].

The concept of the knowledge graph was formally introduced by Google in 2012 [10], with the initial goal of enhancing search efficiency and the capabilities of search engines to improve users’ search experiences. In comparison to traditional knowledge bases, knowledge graphs offer several distinct advantages: Richer semantic relations, knowledge graph can establish the connection between heterogeneous data from multiple sources and use relational information to break the semantic gap between different modal data; higher knowledge quality, the knowledge sources of knowledge graph are diverse, and also provide knowledge representation learning methods such as symbolic representation and vector representation to adapt to different application scenarios; and visualization, knowledge graph supports the visual display, which provides great convenience for human–computer interaction [11]. Currently, knowledge graphs have found widespread application in various industries, including medical, military, financial, and others [12,13,14]. Research on knowledge graphs in the fields of intelligent decision-making, equipment operation, and maintenance has been conducted earlier, and there are now preliminary applications in these areas. Here are some examples: Guo [15] developed a fault diagnosis knowledge graph using text from grid fault disposal plans. This enabled intelligent retrieval of fault information and assisted in diagnosing faults. Guo [16] created a bogie knowledge mapping platform and retrieval system for high-speed trains. It integrated knowledge from multiple sources and domains, aiding designers in product repairability design and improving product quality. Xue [17] constructed a knowledge mapping system for on-board equipment faults, which visually displayed and retrieved relationships between faults. This improved the ability to discover knowledge from on-board fault logs and facilitated fault maintenance guidance. Liu [18] built a service value chain multi-chain knowledge mapping based on third-party cloud platform data resources. This method was validated using relevant service businesses and demonstrated the feasibility of constructing such a knowledge graph. Hu [19] enhanced the traditional knowledge graph construction process by adding text pre-classification and entity reorganization processes. This resolved issues like information redundancy and nested entities present only in text. Liu [20] proposed a fault diagnosis method for mechanical equipment using machine learning algorithms. It accurately predicted fault diagnosis results and used similarity matching to find suitable solutions in the knowledge graph. Mao [21] developed a knowledge graph focused on chemical process safety. It allowed retrieval of potential causes based on safety accident phenomena and ranked corresponding solutions by comparing the probability of potential causes. Wu [22] utilized Bayesian algorithms to mine relationships between ternary groups, enabling knowledge fusion, reasoning, and updating. The resulting knowledge graph facilitated fault prediction, fast discovery, fault localization, type identification, and impact reasoning and provided solutions for decision-making. Li [23] established a comprehensive life cycle knowledge graph for HVDC transmission systems. It combined intelligent decision-making methods based on XGBoost to improve fault diagnosis speed, accuracy, and robustness significantly. Chen [24] proposed a high-voltage substation fault diagnosis method that combined LSTM and knowledge graphs. Integrating fault data with the knowledge graph enabled quick identification and resolution of fault causes, greatly enhancing management, operation, and maintenance efficiency. It is evident that knowledge graphs have been applied in various domains to enhance decision-making, fault diagnosis, and operational efficiency. Pan [25] proposed the Low-Rank Tensor Regularized Graph Fuzzy Learning (LRTGFL) method for processing multiview data. This method clusters the nonlinear structure between multisource heterogeneous data, facilitating a more efficient establishment of relationships between multisource data. Liu [26] utilized a self-supervised approach for knowledge graph completion of multiview data, harnessing the complementarity and consistency of mining multisource heterogeneous data. Consequently, knowledge mapping can yield more comprehensive information by establishing connections between heterogeneous data from multiple sources. This effectively addresses the problem of uncertainty caused by incomplete knowledge in the field of Prognostics and Health Management (PHM) fault diagnosis while also laying the foundation for achieving smarter assisted maintenance decisions.

Currently, there is limited integration of knowledge graphs in the field of aircraft Prognostics and Health Management (PHM). However, the combination of knowledge graphs with aircraft PHM holds significant potential for broad applications and high utility. While research on knowledge mapping has made some headway in the realm of aircraft manufacturing [11], its application in aircraft PHM is still in its early stages. For example, Zhang [27] introduced the BERT-BiLSTM-CRF algorithm for identifying defective entities in aero-engines but did not provide further details on the construction method for the aero-engine fault knowledge graph. In a similar vein, Nie [28] proposed key technologies for constructing knowledge graphs related to aircraft power system fault diagnosis and conducted exploratory applications of intelligent systems. Additionally, Wu [29] utilized the BERT-BiLSTM-CRF algorithm for entity relationship extraction within the aero-engine lubrication system, subsequently constructing a fault knowledge graph for this system. Tang [30] employed deep learning as the primary method and heuristic rules as an auxiliary method to extract fault knowledge from both structured and unstructured fault data, leading to the construction of a fault knowledge graph for a specific type of process. Furthermore, Meng [31,32] presented a method for constructing a fault knowledge graph for aircraft power system health management and developed an intelligent Q&A system based on this knowledge graph, resulting in a significant enhancement of maintenance personnel’s troubleshooting abilities.

The research aims to integrate knowledge graph technology into aviation fault diagnosis. Although these efforts have improved the efficiency of constructing fault knowledge bases and the correlation performance of faults, they may not fully address the semantic complexity of unstructured text during the knowledge graph construction process. In this paper, we introduce a knowledge graph construction process specifically tailored for aircraft health management, focusing on the flight control system as an illustrative example. Additionally, we developed a knowledge graph management platform dedicated to aircraft health management, facilitating the rapid construction of knowledge graphs and Q&A systems.

Compared with previous work, this paper presents the following key contributions and innovations:

(a) Proposal of a knowledge graph construction process for aircraft health management: The paper introduces a tailored process to meet the specific requirements of aircraft health management. To address the complexities and ambiguities of text semantics in entity relationship extraction, the approach incorporates text classification to classify different segments of the text, thereby reducing the difficulty of entity relationship extraction.
(b) Evaluation of a pre-trained linguistic joint extraction model based on ERNIE: The paper assesses the effectiveness of a pre-trained linguistic joint extraction model leveraging ERNIE. This model can generate feature vectors and encode contextual semantic information. By integrating LSTM based on sequences and tree structures, as well as syntactic dependency trees, the model effectively captures the interdependence between entities and relations. This approach reduces error transmission in entity-relationship extraction and mitigates the semantic complexity of the text.
(c) Development of a knowledge graph construction platform for aircraft health management: The paper introduces a comprehensive platform equipped with modules such as text classification, knowledge extraction, knowledge audit, Q&A systems, and graph visualization. This platform enhances the management capabilities of aircraft health management data and provides a solid foundation for the rapid construction of aircraft health management knowledge graphs.

These contributions represent significant advancements in the field of aircraft health management and knowledge graph construction, addressing the limitations of previous research endeavors.

## 2. Data Sources and Analyses

### 2.1. Data Sources

The data utilized in this paper comprise flight control system manuals and typical failure cases related to a specific type of aircraft. Table 1 presents the data nomenclature and types employed in this study. Upon observation, it becomes apparent that the system manual primarily consists of unstructured data, including function descriptions and composition descriptions. Similarly, typical failure cases encompass unstructured data such as failure phenomena and cause of failure. The objective of this paper is to extract the flight control system triad from the system specifications and typical failure cases, subsequently leveraging this information to construct an aircraft health management knowledge graph.

### 2.2. Data Analysis

The text within the system manual and typical failure cases of the flight control system analyzed in this paper exhibit the following characteristics:

(a) Abundance of specialized nouns: The text contains a substantial number of specialized nouns, making direct employment of a thesaurus problematic due to the potential for inaccurate entity extraction results.
(b) Similar semantic structure across diverse data sources: While the semantic structure of the text is comparable across various sources, the extraction model encounters challenges due to differing extraction content, leading to potential confusion.
(c) Complexity of data content: The data encompass complex content wherein certain texts necessitate the entire sentence as an entity, while other texts do not require the extraction of entity relations. This variance significantly impacts the efficacy of ternary entity relation extraction.

To address the aforementioned characteristics, this paper proposes the following solutions for constructing the knowledge graph in aircraft health management: Problem 1: Specialized Nouns This paper incorporates domain-specific nouns into the entity extraction model using a dictionary. This approach prevents the influence of specialized nouns on the extraction process, thereby improving extraction accuracy. Problem 2: Semantic Structure Differences Before performing entity relationship extraction, this paper employs a text classification process. By doing so, it mitigates the impact of semantic structure variations across different data sources on the extraction model, enhancing its adaptability. Problem 3: Text Classification Information The paper introduces text classification information as a gating mechanism within the entity-relationship extraction model. It leverages global text classification data to determine the extraction mode of the text, effectively avoiding the influence of special text content on normal text triad extraction.

## 3. Knowledge Graph Construction for Aircraft Health Management

### 3.1. Knowledge Graph Construction Framework

**Figure 1:**
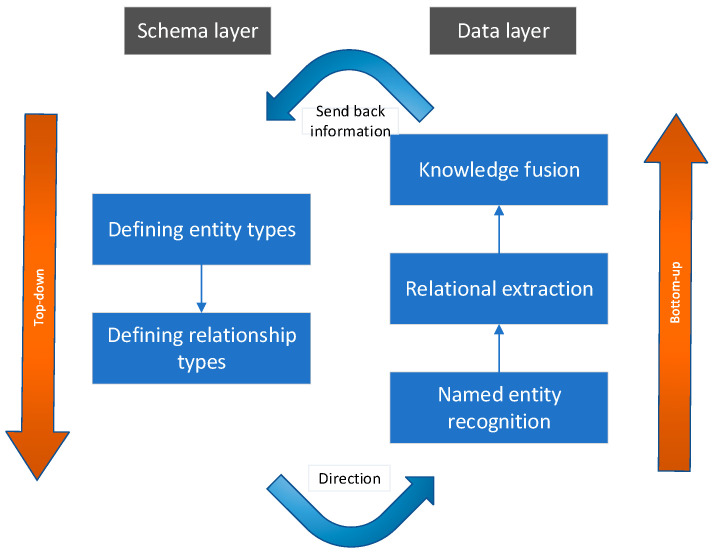
Knowledge Graph Construction Process.

There are three primary construction frameworks for knowledge graphs: top-down, bottom-up, and a combination of both [33]. In the bottom-up approach, knowledge extraction precedes the definition of ontology information, while the top-down approach involves defining ontology information first, followed by knowledge extraction from the data. Currently, domestic domain knowledge graph constructions predominantly employ the top-down method. However, given the diverse data sources and schema disparities within the aircraft health management domain, this paper advocates for a combined top-down and bottom-up construction approach. Specifically, this approach entails initially defining entity types and relationship types in the schema layer to guide the establishment of the data layer. Subsequently, the data layer is updated during the establishment process, continuously providing feedback to the schema layer. The construction process is illustrated in Figure 1.

### 3.2. Construction of the Schema Layer

**Figure 2:**
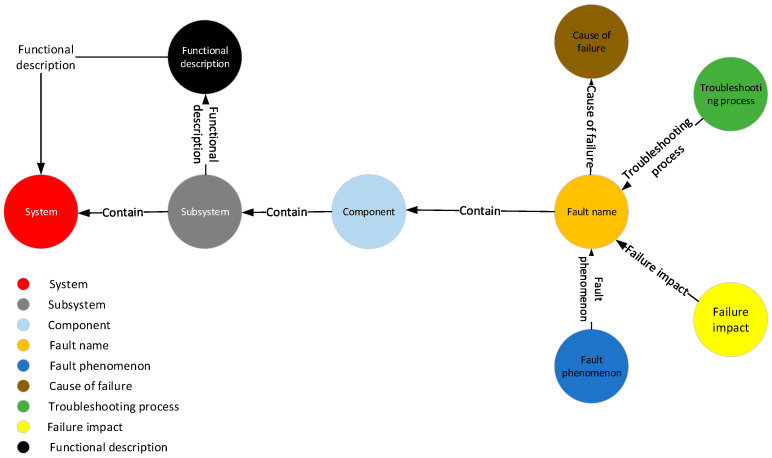
The schema layer of knowledge graph.

The schema layer of the knowledge graph serves as a semantic specification, guiding the construction of the data layer and providing a semantically classified framework within a specific scope. In this paper, the schema layer of the aircraft health management knowledge graph, depicted in Figure 2, primarily encompasses nine entity types, such as parts, faults, fault phenomena, and causes of faults, along with seven relationships between these entity types.

### 3.3. Construction of the Data Layer

The construction of the data layer primarily involves three tasks: entity extraction, relationship extraction, and knowledge fusion. Among these, entity extraction serves as the foundation for knowledge graph construction. It is also known as named entity recognition (NER), and its primary objective is to extract entities from unstructured data. Relationship extraction, on the other hand, is a crucial subtask of knowledge graph construction that focuses on extracting semantic relationships between two entities from unstructured data. Additionally, there may be cases where extracted entities have multiple meanings or are near-synonyms, requiring knowledge fusion processing. This involves entity disambiguation and co-reference disambiguation tasks.

Given that the entities in semi-structured data lack contextual information, this paper adopts a manual approach to carry out knowledge fusion between semi-structured data and unstructured data.

#### 3.3.1. Text Classification

The processing result field in a typical fault case comprises four main contents: fault phenomenon, troubleshooting process, cause of fault, and fault impact, as outlined in Table 2.

In a typical fault case, each part shares semantic similarities, yet their extraction methods differ. For instance, in the troubleshooting process, each sentence should be treated as an entity, while in other parts, the model needs to extract entities and relationships from the text. Additionally, there are instances where certain texts do not require entity and relationship extraction. These varied scenarios introduce ambiguity for the model. Directly performing entity extraction on the processing result field would heighten the extraction complexity due to the presence of other categories of information. As a result, the text content in the field is considered to be pre-categorized, constituting a text categorization task.

**Figure 3:**
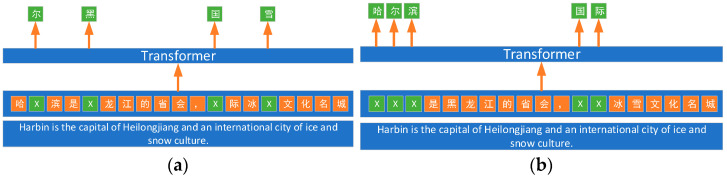
Pre-training model approach (**a**) BERT pre-training method; (**b**) ERNIE pre-training method.

In current related research, machine learning models or deep learning models are commonly employed for text classification. The model utilized in this paper is the ER-NIE model, which is based on the transformer architecture proposed by Sun [34]. The ERNIE model, introduced by Baidu, is a pre-trained language model that leverages knowledge enhancement, endowing it with a robust ability to learn text representations. Specifically, the ERNIE model facilitates the learning of semantic representations for complete concepts by masking semantic units such as words and entities. In comparison to BERT, which learns raw linguistic signals, ERNIE directly models a priori semantic knowledge units, thereby enhancing the model’s semantic representation capability. The pre-training approach of the ERNIE model and BERT model is illustrated in Figure 3.

#### 3.3.2. Joint Extraction of Entity Relationships Based on ERNIE-BiLSTM-CRF-TreeBiLSTM

Entity-relationship joint Extraction refers to the task of simultaneously identifying and extracting entities along with the relationships between them in a given text. In contrast to traditional named entity extraction, which solely focuses on entity identification, entity relationship joint extraction aims to achieve a more comprehensive understanding of the semantic associations between entities present in the text.

The traditional extraction approach involves initially conducting entity recognition within the sentence and then determining the type of relationship between entities based on the combination of subject and object in the entity extraction result, thereby forming a ternary group of subject, relationship, and object [35]. Nevertheless, this extraction approach has certain shortcomings. Firstly, it faces the issue of error accumulation, where any errors in entity recognition will consequently impact the subsequent relationship classification results, leading to further error propagation [36]. Secondly, there is an underutilization of information, as the tasks of entity extraction and relationship extraction are relatively independent, and the inherent connection between the two is not effectively leveraged, particularly with relationship extraction failing to capitalize on the relationship between the two [37]. To mitigate these challenges, a joint relational extraction model can be employed. This model is designed to maximize the potential information between the two tasks and enhance the interaction between the entity recognition model and the relationship classification model, thereby addressing these limitations [38].

Amidst the emergence of the big data era, numerous scholars have dedicated themselves to the exploration of entity-relationship joint extraction. For instance, Zheng [39] devised a new annotation scheme to transform the entity-relationship joint extraction task into a sequence annotation problem. Sun [40] employed graph convolutional neural networks for relationship extraction, utilizing relationship weights to capture connections between multiple entity types and relationship types within a sentence. Nayak [41] introduced a joint entity-relationship extraction method utilizing an encoder-decoder architecture, leading to a substantial enhancement in the F1 score. Yu [42] proposed a novel entity-relationship extraction strategy using Span’s annotation scheme to break down the joint extraction task into two interconnected subtasks (HE extraction and TER extraction), which exhibited noteworthy performance improvements in test application scenarios. Wei [43] put forward a new cascading binary annotation framework, Casrel, which translates the task of extracting triples into three levels: central entities, relations, and object entities. Katiyar [44] was the first to introduce the attention mechanism in combination with a bi-directional LSTM for joint entity-relationship extraction. In the literature [45], the attention mechanism for neural networks in relation to extraction was introduced, proving to be more effective than other structured perceptron joint models. Literature [46] proposed an entity-relationship extraction model based on the sequence and tree structure of LSTM and integrated syntactic dependency trees to reduce the linguistic complexity of utterances, offering valuable insights and ideas for further research and problem-solving.

**Figure 4:**
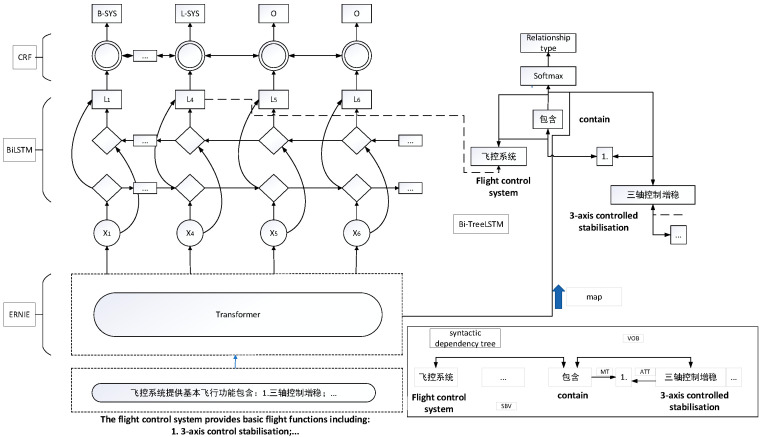
Entity-relationship extraction model based on ERNIE-BiLSTM-CRF-TreeBiLSTM.

This study employs a classical end-to-end relationship extraction model based on sequence and tree structure LSTM. The model uses an end-to-end neural network structure to extract entities and entity relationships by leveraging both sequence and dependent tree structure information. To enhance the parsing ability of the sequence and tree-structured LSTM model, we introduce the ERNIE model as the embedding layer of our model. The output of the ERNIE model is connected to the sequence and tree-structured LSTM model. Additionally, we incorporate the output vector X from text classification based on the ERNIE model into the relationship extraction task. This helps address the impact of special text on relationship extraction. The entity-relationship extraction model, known as ERNIE-BiLSTM-CRF-TreeBiLSTM, is illustrated in Figure 4.

The specific process is as follows: first, the text sequence’s lexicality is annotated using the syntactic dependency tree. In this paper, the Baidu-DDParser tool is utilized for the syntactic analysis of the text. Subsequently, the text is converted into a text sequence. The input vector X of the ERNIE model is then transformed into the ERNIE model, as depicted in Equation (1).

(1) $$\left\{ \begin{array}{l} X=\left[x_{1}\;x_{2}\;\ldots \;x_{n}\right]\in R^{n\times H}\\ x_{i}=E_{i}^{t}+E_{i}^{p}+E_{i}^{s}\;i=1,2,\ldots ,n \end{array}\right.$$

The process starts with *H* representing the vector dimension, $E_{i}^{t}$ denoting the word embedding coding for word sequences, and $E_{i}^{p}$ representing the positional information coding for word sequences. Additionally, $E_{i}^{s}$ signifies the utterance information coding for word sequences. These components are collectively mapped to attain the input sequence *X* of the ERNIE layer through high-dimensional summation.

Subsequently, the sequence *X* undergoes the computation of comprehensive features via the multilayer Transformer to yield the final feature vector *T*. This feature vector *T* is then fed into the Sequential BiLSTM layer, which captures linguistic features based on contextual information, thereby producing a sequence of vectors $O=\left[o_{1}\;o_{2}\;\ldots \;o_{n}\right]$ that integrates contextual details. Following this, a sequence of vectors *O* is generated and input to the CRF layer.

Ultimately, the CRF layer derives the optimal entity labels, employing the globally best label sequence based on neighboring label relationships. This process yields the optimal entity labels labeled with BILOU (Begin, Inside, Last, Outside, Unit).

In order to incorporate both the word sequences and dependency tree structure information into the output, we introduce the dependency layer on top of the sequence layer. The LSTM unit within the dependency layer receives $x_{t}=[s_{t};v_{t}^{\left(d\right)}]$ as input at the t-th word, and its corresponding hidden state vector $s_{t}$ is then integrated with the sequence layer, specifically with the sequence BiLSTM embedding $v_{t}^{\left(d\right)}$.

The TreeLSTM produces three outputs: $d_{p}$ = [$h_{p_{A}}$; $h_{p_{1}}$; $h_{p_{2}}$; $h_{p_{E}}$] represents the hidden state vector of the top LSTM cell in the bottom-up TreeLSTM, while $h_{p_{A}}$, $h_{p_{1}}$, and $h_{p_{2}}$ denote the hidden state vectors of the two LSTM cells representing the first and second target words in the top-down TreeLSTM. Additionally, $h_{p_{E}}$ signifies the hidden state vector of the last word in the ERNIE model. These associations are depicted by the corresponding arrows in Figure 1. Finally, a two-layer neural network, as depicted in Equation (2), comprising a $n_{h_{r}}$ dimensional hidden layer $h^{\left(r\right)}$ and a softmax output layer, is employed to output the relationship type.

(2) $$h_{p}{}^{\left(r\right)}=\tanh(W^{\left(r_{h}\right)}d_{p}+b^{\left(r_{h}\right)})\;y_{p}=\mathrm{softmax}(W^{\left(r_{y}\right)}h_{t}{}^{\left(r\right)}+b^{\left(r_{y}\right)})$$

where $W^{\left(r_{h}\right)}$ and $W^{\left(r_{y}\right)}$ are the weight matrix, $b^{\left(r_{h}\right)}$ and $b^{\left(r_{y}\right)}$ are the bias vector.

## 4. Test Analyses

### 4.1. Test Platform Construction and Test Indicators

We labeled 1648 entities from a total of 1000 faulty texts, with 700 texts allocated to the training set and 300 texts assigned to the test set. The evaluation metrics for joint extraction of entity relations include accuracy, as depicted in Equation (3), recall, as depicted in Equation (4), and *F*_1_ value, as depicted in Equation (5). The *F*_1_ value serves as a comprehensive evaluation metric for assessing the model’s extraction effectiveness and is calculated using the following formula:

(3) $$P=\frac{T_{p}}{T_{p}+F_{p}}$$

(4) $$R=\frac{T_{p}}{T_{p}+F_{N}}$$

(5) $$F_{1}=\frac{2PR}{P+R}$$

In the experiment, the evaluation metrics are defined as follows: *T_p_* represents the positive examples that were correctly predicted, *F_p_* denotes the positive examples whose true value was a counterexample that was incorrectly predicted, and *F_N_* refers to the positive examples whose true value was incorrectly predicted as a counterexample. The joint extraction model constructed in this paper considers a triad to be correctly predicted when the head entity, relationship entity, and tail entity are all predicted accurately.

### 4.2. ERNIE-Based Text Classification

Text can be broadly categorized into two groups: normal text and special text. Normal text includes fault causes, fault phenomena, fault effects, and troubleshooting processes. On the other hand, special text can be further divided into two subcategories: whole-sentence extraction and non-extraction. Prior to training the model, it is necessary to configure the model parameters. The training parameters for the text classification model are outlined in Table 3. During training, the iteration is stopped when the difference in accuracy between the latest 10 iterations is less than 0.2.

**Figure 5:**
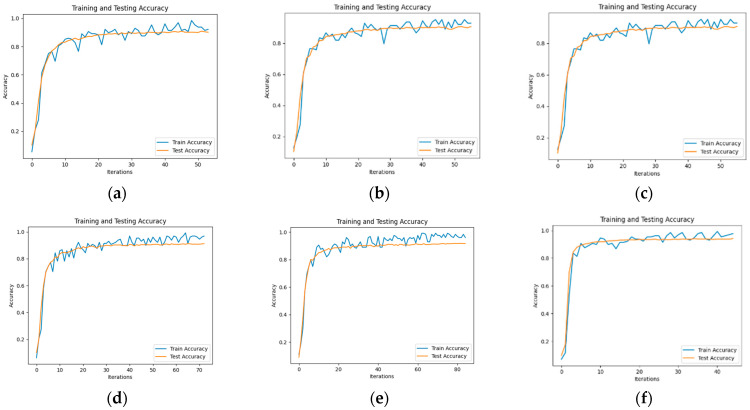
Model Text Classification Accuracy: (**a**) BERT Model Text Classification Accuracy; (**b**) BERT-CNN Model Text Classification Accuracy; (**c**) BERT-RNN Model Text Classification Accuracy; (**d**) BERT-RCNN Model Text Classification Accuracy; (**e**) BERT-DPCNN Model Text Classification Accuracy; (**f**) ERNIE Model Text Classification Accuracy.

To evaluate the performance of the text classification model presented in this paper, we conducted a comparative test using several models, including BERT [47], BERT_CNN [48], BERT_RNN, BERT_RCNN, and BERT_DPCNN [49]. The model accuracy diagram is illustrated in Figure 5, and the test results are summarized in Table 4, where the test results are taken as the average of 5 tests, and the *F*_1_ values are accompanied by upper and lower limits.

**Figure 6:**
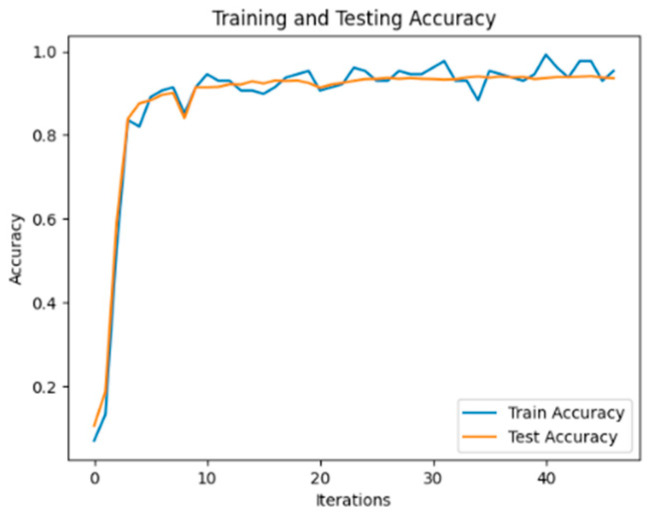
ERNIE_RCNN Model Text Classification Accuracy.

The experimental results demonstrate that, on the flight control system fault dataset developed in this study, the ERNIE model outperforms other benchmark models in terms of precision, recall, and *F*_1_ score values when it comes to classifying each part of the text in a typical fault case. This superiority can be attributed to the utilization of word pairs in the form of masking during the pre-training task, which maximizes the extraction of textual information. Additionally, in the model using BERT as the embedding layer, BERT_RCNN exhibits better classification performance. This indicates that the BiLSTM module can effectively capture semantic information from the text. To explore this aspect further, another experiment involving the ERNIE_RCNN model was conducted. As depicted in Figure 6, the accuracy value of the ERNIE_RCNN model reaches 94.75%. Therefore, this paper selects the ERNIE_RCNN model as the text classification model for typical fault cases in the flight control system.

### 4.3. Test on Joint Extraction of Entity Relationships Based on ERNIE-BiLSTM-CRF-TreeBiLSTM

Before model training, the model parameters need to be set; the training parameters of the ERNIE-BiLSTM-CRF-TreeBiLSTM model are shown in Table 5.

To verify the superiority of the model ERNIE-BiLSTM-CRF-TreeBiLSTM to the fault triple extraction task, this paper selects the mainstream extraction models, such as Casrel [43] and GPlinker [50], as the control and conducts comparative experiments on the self-built flight control system fault data set. The experimental results are shown in Table 6, where the test results are taken as the average of 5 tests, and the *F*_1_ values are accompanied by upper and lower limits.

The experimental results demonstrate that the ERNIE-BiLSTM-CRF-TreeBiLSTM model, applied to the flight control system fault dataset constructed in this study, effectively addresses the challenge of semantic complexity in PHM domain text. It significantly enhances ternary extraction, yielding better precision, recall, and *F*_1_ score values compared to other benchmark models. Furthermore, a comparison between ERNIE-BiLSTM-CRF-TreeBiLSTM*, Casrel, and GPlinker reveals that the ERNIE model outperforms the BERT model in mining Chinese text information. Additionally, comparing the ternary extraction effects of ERNIE-BiLSTM-CRF-TreeBiLSTM* with ERNIE-BiLSTM-CRF-TreeBiLSTM demonstrates that categorizing text between ternary extraction improves the effectiveness of handling the semantic complexity of text during ternary extraction. By utilizing ERNIE-BiLSTM-CRF-TreeBiLSTM for entity-relationship recognition within ternary groups, the proposed model in this study effectively enhances the efficiency of extracting ternary group information. It strengthens the correlation between entity recognition and relationship extraction, facilitating the integration and utilization of fault information. This advancement provides valuable technical support in the field of fault diagnosis.

## 5. Design and Implementation of Knowledge Graph Platform

### 5.1. Platform Architecture Design

**Figure 7:**
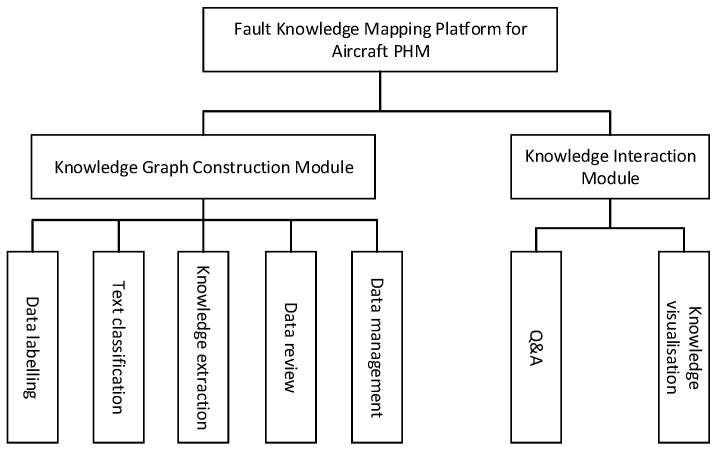
System Functional Module Diagram.

To streamline data management and expedite the development of Prognostics and Health Management (PHM)-oriented knowledge graphs, this paper introduces a fault knowledge graph platform tailored for aircraft PHM. Figure 7 illustrates the design of the platform’s functional modules, encompassing the system’s overall architecture and the interconnections among each functional module.

Figure 7 presents the functional modules of the knowledge mapping platform for aircraft Prognostics and Health Management (PHM) faults. It primarily consists of a knowledge map construction module and a knowledge interaction module. The data annotation function is utilized to annotate ternary data and provide training samples for the knowledge extraction model. The text classification function performs sentence-level classification, reducing the impact of semantic complexity between different texts on subsequent knowledge extraction. The knowledge extraction function extracts entity types and relationship types from the text. The data audit function allows manual auditing of the ternary extraction effect, enabling modification of the results. The data management function handles user-uploaded data, encompassing equipment fault statistics, equipment structural information, and equipment status visualization. The intelligent Q&A function and knowledge visualization function facilitate human–computer interaction by providing feedback to user queries and presenting information visually.

**Figure 8:**
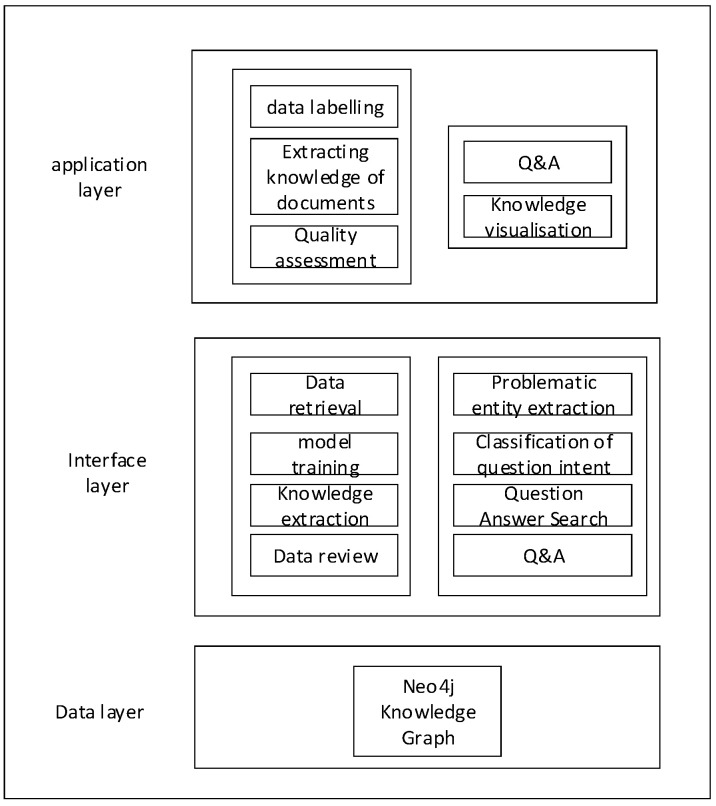
System Functional Architecture Diagram.

The system’s functional architecture, depicted in Figure 8, comprises the application layer, interface layer, and data layer from top to bottom. The application layer is user-centric, empowering the administrator user to conduct data annotation, extract document knowledge, and perform quality assessments within the front-end. Additionally, the knowledge interaction user can intelligently pose and respond to questions, as well as engage in knowledge visualization. Meanwhile, the interface layer serves to actualize the system’s functions, encapsulating the implementation details and serving as the system’s core. Lastly, the data layer primarily encompasses the Neo4j knowledge graph within this system. The specific details of the architecture of the system functionality are described in Section 5.3 and Section 5.4 of this paper.

### 5.2. Platform Development Environment and Tools

Table 7 demonstrates the development environment for this experiment, including the hardware environment and software environment.

The system’s front-end is developed using Vue, a modern JavaScript framework designed for constructing user interfaces. Vue aims to assist developers in creating interactive, responsive single-page applications (SPAs) by offering user-friendly APIs and efficient rendering performance. On the other hand, the back-end is powered by FastAPI, a contemporary and swift Python-based web framework tailored for building APIs.

### 5.3. Knowledge Graph Building Module

#### 5.3.1. Design of Knowledge Graph Building Module

**Figure 9:**
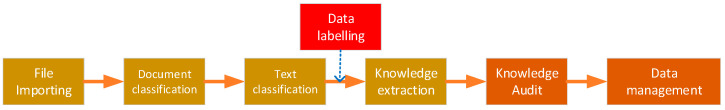
Knowledge Graph Building Module Design Process.

Figure 9 illustrates the design flow of the knowledge graph-building module, encompassing file import, file classification, text classification, data extraction, data annotation, data audit, and data management. Notably, text classification and knowledge extraction are automatically executed by algorithms in the background. Consequently, the subsequent focus is primarily on implementing the data labeling module, knowledge audit module, and data management module.

#### 5.3.2. Data Labeling Module

**Figure 10:**
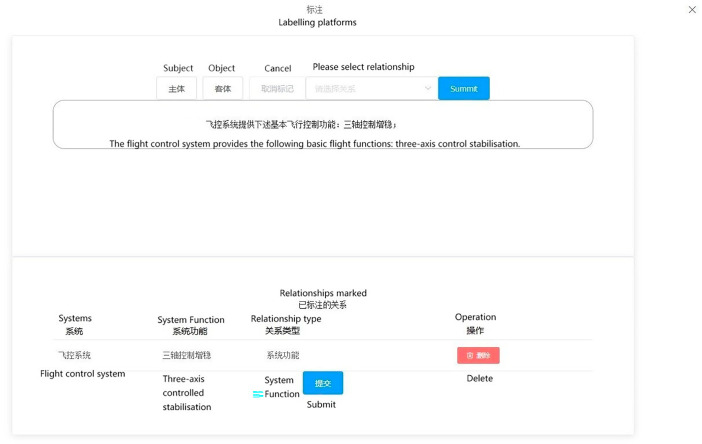
Data labeling interface.

The data annotation process is primarily triggered when a new document type is imported, enabling users to annotate the newly uploaded text. The platform conducts annotations based on the outcomes of text classification illustrated in the diagram. The primary objective involves annotating entity types, subjects, objects, and the relationships between subjects and objects within the text. Once all texts are annotated, they are transmitted to the back-end to furnish data support for the knowledge extraction model. The output of the data annotation interface is depicted in Figure 10.

To implement front-end annotation highlighting, the Tiptap rich text editor plugin can be employed. Tiptap is a Vue-based rich text editor that utilizes a JSON data structure for managing text content and styles. To begin, initialize the text editor using the Editor component provided by Tiptap. Then, utilize the highlight() method to achieve text highlighting. This method allows you to specify the selection and apply a highlight to the desired portion of text. Additionally, you can use the selection feature to obtain the text content and position of the selected words or phrases. By leveraging Tiptap’s functionalities, you can effectively enable annotation highlighting within the front-end interface.

The system consolidates the annotation results into a JSON string, which is then transmitted to the back-end and can be saved by the user. This JSON string encompasses the sentence ID, the original text content, and the triple spo_list. The spo_list includes the entity type, entity location, and relationship information for effective data representation and storage. For example: {“ID”: “AT0000”, “text”: “The flight control system provides the following basic flight functions: three-axis control stabilisation”, “spo_list”: [{“h”: {“ System “: “Flight Control System”, “pos”: [0, 4]}, “t”: {“ System Function “: “Three-Axis Control Stabilisation”, “pos”: [16, 21]}, “relation”: “System Function”}]}.

#### 5.3.3. Data Review Module

**Figure 11:**
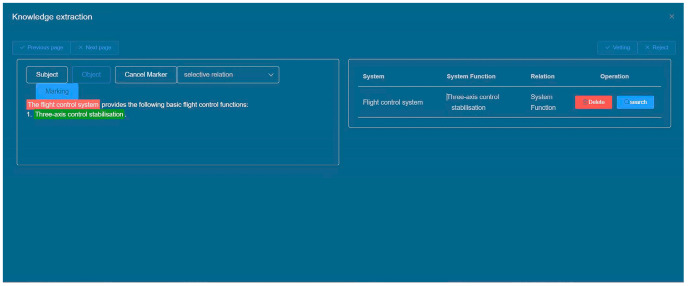
Data Review Interface.

The data review interface enables users to inspect the knowledge extraction results, identify the original text location, and subsequently rectify any erroneous extraction outcomes while also adding new entities and relationships as necessary. Figure 11 provides a visual representation of the data review interface.

**Figure 12:**
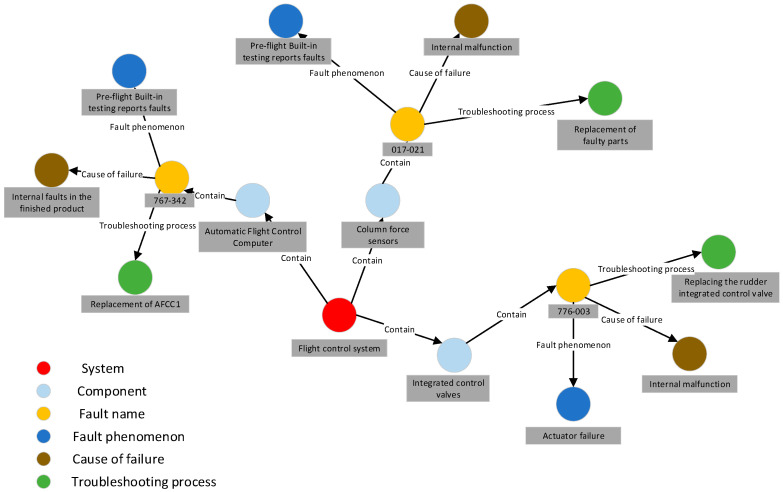
Partial Knowledge Graph Presentation.

Once the data review process is finalized, the ternary results can be imported into the Neo4j graph database to construct a knowledge graph. Figure 12 presents a portion of the resulting knowledge graph.

#### 5.3.4. Data Review Module

The primary role of the data management module is to facilitate the centralized management of data. This includes storing information such as past failure cases and data related to the composition structure of each component within the system. The data management module ensures efficient organization, retrieval, and maintenance of these diverse sets of data.

**Figure 13:**
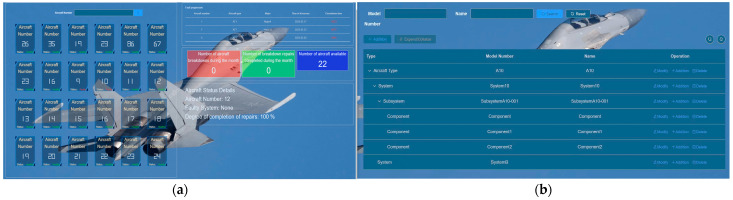
Data Management Interface: (**a**) The aircraft status overview interface; (**b**) Equipment information interface.

As illustrated in Figure 13, the data management module plays a crucial role in the PHM system by providing unified management of heterogeneous data from multiple sources. Figure 13a showcases the aircraft status overview interface, which offers a comprehensive view of aircraft status data. This includes information on aircraft failures, troubleshooting progress, and historical failure records. Users can easily access and analyze this data using the aircraft status overview map. Figure 13b presents the equipment information interface, enabling users to visualize the structural composition of data between different aircraft systems. This map allows for a clear understanding of how data is interconnected and provides insights into the relationships and dependencies among various components. By facilitating the effective management and organization of diverse data, the data management module establishes a solid foundation for the PHM system’s operations, enhancing its ability to handle data from multiple sources efficiently and cohesively.

### 5.4. Knowledge Interaction Module

#### 5.4.1. Design of the Knowledge Interaction Module

The Knowledge Interaction Module typically involves several steps, including entity recognition, question type identification, knowledge matching, and answer generation. However, in this experiment, the question parsing part is not required as it utilizes its own model for entity recognition. The principle of entity recognition remains the same as the entity recognition employed in the previous knowledge extraction phase, so there is no need to repeat it.

**Figure 14:**
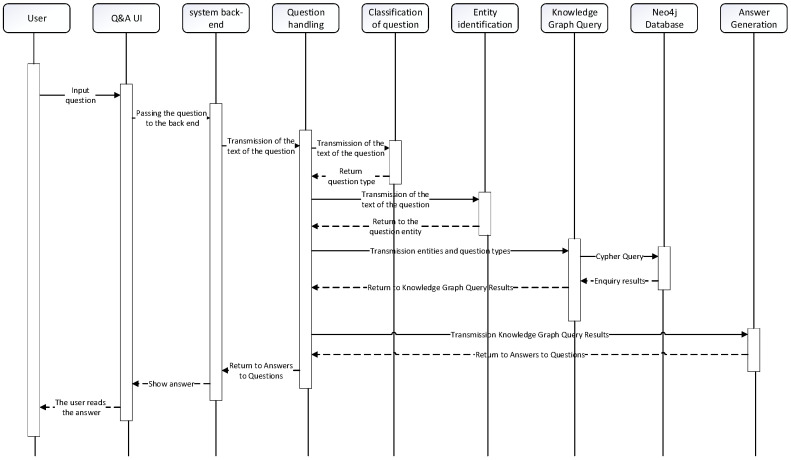
Intelligent Question and Answer Sequence Chart.

**Figure 15:**
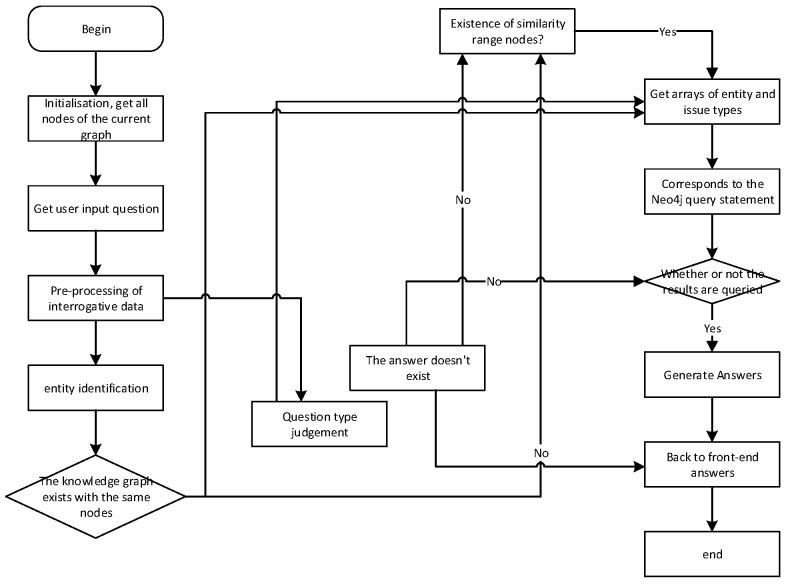
Flowchart of Intelligent Q&A.

Figure 14 showcases the sequence diagram of the natural language question and answer module. The process begins with the user inputting a question, which is then passed from the front-end to the back-end. The question classification part identifies the question type using user intent classification labeling tags and keyword lists. Next, in the entity identification part, the entity within the question is determined. Once the question’s entity and category are obtained, they form the basis for constructing a Cypher query statement. This Cypher query statement is then passed to the Neo4j graph database. The Neo4j graph database processes the query and returns an array of Answers. Based on the question’s category, an answer generation template is used to format the array of answers into a natural language form. This formatted answer is returned to the front-end and presented to the user for reading. Figure 15 showcases the flowchart of the smart Q&A system, providing a visual representation of the overall process.

#### 5.4.2. Q&A Module

The connection between the Python back-end and the Knowledge Graph is established via the utilization of the py2neo library. Py2neo serves as a driver for the Neo4j graph database specifically designed for Python. It offers a user-friendly API that facilitates seamless interaction with Neo4j databases, enabling various query and modification operations to be performed efficiently.

**Figure 16:**
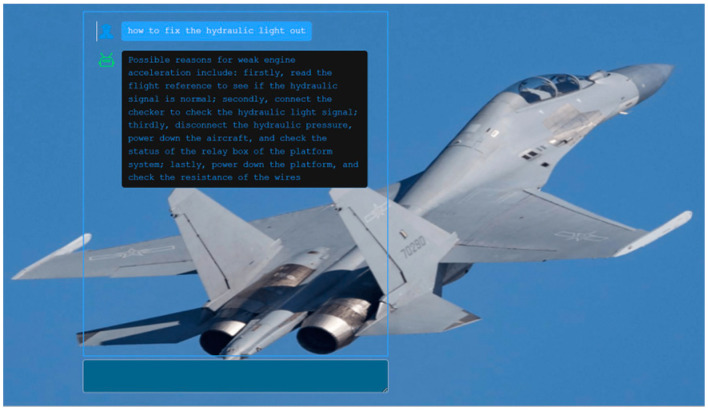
Intelligent Q&A interface.

During initialization, the system will read the nodes in the graph to enable subsequent similarity calculations. After the user inputs a question and submits it, the normal question-and-answer process is executed. This involves attempting to successfully identify the relationship between the target entity and the corresponding entity in the graph. If the relationship is successfully found, the answer stored in the graph will be provided in a natural language format. However, if the entities extracted from the question do not have a corresponding node within the similarity threshold, the answer will be “no corresponding node exists at all”. In cases where nodes are found but there is no corresponding relationship between them, the answer will be “there is no [relationship] for [entity]”. The implementation of intelligent Q&A is demonstrated in Figure 16, which illustrates the resulting outcome.

In Figure 16, the user inputs the question “how to fix the hydraulic light out”, the front-end passes the question to the back-end, and the back-end determines that the type of the question is “troubleshooting process” based on “how to fix” in the sentence. The entity extraction part extracts the entity “hydraulic light is out”, the combination result and the node “hydraulic light does not light up” in the graph have the closest similarity, and the entity type of the question is “hydraulic light does not light up”, according to the entity type of the question “hydraulic light does not light up”, the entity type of the question is “hydraulic light does not light up”. The entity type of the question is “hydraulic light is not on”, and according to the entity and the type of the question, the Cypher query statement Cypher statement “MATCH (n {name: ‘Hydraulic light is not on’})-[r1:Relation {name: ‘Troubleshooting process’}]->(m)RETURN n.name, m.name” is generated, which can be obtained as the return result [{‘Hydraulic light is not on’}]->(m)RETURN n.name, m.name”. Result[{‘n.name’: ‘Hydraulic light is not on’, ‘m.name’: ‘Read the flight reference, make sure the hydraulic signal is normal’}, {‘n.name’: ‘Hydraulic light is not on’, ‘m.name’: ‘Connect the checker, check the hydraulic light signal’}, {‘n.name’: ‘Hydraulic light is not on’, ‘m.name’: ‘Disconnect the hydraulic pressure, power down the plane., check platform system relay box status’}, {‘n.name’: ‘Hydraulic light not on’, ‘m.name’: ‘Platform powered down, check wire resistance’}]. The result is passed to the intelligent answer generation section, and the “troubleshooting process” corresponds to the template. Finally, the answer “Possible reasons for weak engine acceleration include: firstly, read the flight reference to see if the hydraulic signal is normal; secondly, connect the checker to check the hydraulic light signal; thirdly, disconnect the hydraulic pressure, power down the aircraft, and check the status of the relay box of the platform system; lastly, power down the platform, and check the resistance of the wires” is generated and passed to the front-end display. The answer to the question was passed to the front-end display.

#### 5.4.3. Knowledge Presentation Module

The Knowledge Presentation module utilizes Neovis.js to establish a connection between the Vue front-end and the Neo4j graph. Neovis.js is a JavaScript library specifically designed for visualizing Neo4j graph databases within a web browser. It offers a straightforward API that simplifies the process of creating interactive and customizable graphical visualization interfaces. This allows users to easily build visually appealing and interactive representations of the data stored in the Neo4j graph.

To enable specific knowledge search and display, the input provided in the input box is transformed into a Cypher statement to construct a query. The results of this query are then visually presented to the user, allowing them to interact with the displayed information. Users have the capability to drag nodes and zoom in and out within the interface, facilitating ease of viewing. Furthermore, adjustments were made to the coefficients to display the name attribute of entities and relationships, as well as to illustrate the connections between relationships. This enhancement improves the clarity and comprehensiveness of the visual representation, thereby enhancing the user’s experience while exploring the knowledge graph.

**Figure 17:**
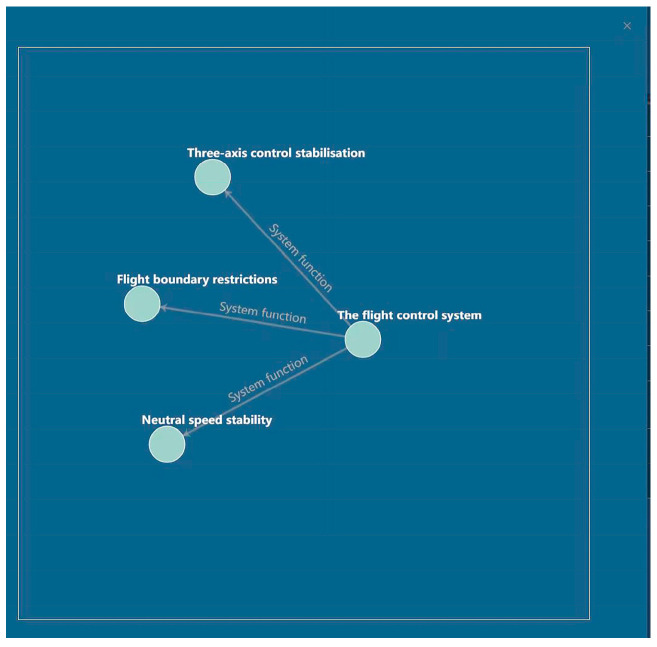
Knowledge Visualisation interface.

For instance, when the user inputs “ flight control system “, Neo4j performs a Cypher query with the statement “MATCH (n:Entity {name: ‘engine’})-[r]->(m) RETURN n, r, m”. This query retrieves the node “engine” along with the nodes and relationships connected to it. The front-end processes this data using Neovis.js and displays the visual representation accordingly. In the event that no corresponding node is found in the search result, a pop-up message “No corresponding node exists” is displayed to notify the user. The knowledge display interface is illustrated in Figure 17.

## 6. Conclusions and Discussion

This paper proposes a method for constructing a fault knowledge graph specifically for aircraft Prognostics and Health Management (PHM) applications. The method addresses the unique requirements of the aircraft PHM domain. The process begins with text classification based on the characteristics of aircraft PHM data. This step aims to reduce the complexity and ambiguity of Chinese text, making it easier for subsequent knowledge extraction. Next, a joint extraction model for entity relations is introduced. This model tackles the challenge of error transmission during the extraction of entity relations while also reducing the linguistic complexity of the text from a semantic perspective. The proposed method is then applied to construct the fault knowledge graph for a specific aircraft flight control system. The resulting knowledge graph is visually displayed, providing an intuitive representation of the information. Finally, a fault knowledge graph management platform for aircraft PHM is developed. This platform facilitates the rapid construction of knowledge graphs, unified management of multisource heterogeneous data, knowledge visualization, and intelligent Q&A capabilities.

Experience has demonstrated that knowledge graph technology can effectively leverage diverse sources of heterogeneous fault data within the PHM domain to establish correlations among fault data. By distilling insights from historical data, knowledge graphs offer substantial potential for enhancing fault diagnosis within the PHM domain. Nevertheless, it is important to acknowledge the limitations of this study, which warrant further exploration in the future.

The knowledge graph presented in this paper is centered on textual data. However, fault data gathered within the PHM domain typically comprises diverse, multisource heterogeneous content, including images, videos, and signals. Establishing semantic associations across such varied data types poses a significant challenge. Consequently, to tackle this issue, our future work will entail conducting a thorough investigation into multimodal knowledge graphs.

This paper leverages traditional knowledge graphs to address the challenge of fault diagnosis within the PHM domain. However, conventional knowledge graphs primarily establish static representations, overlooking the dynamic relationships between fault entities during the fault diagnosis process. Effectively realizing the dynamic association between entities within the fault knowledge graph is pivotal for laying the groundwork for fault prediction in the PHM domain—a task ripe for significant research exploration. Consequently, we aim to incorporate time information into the knowledge graph to facilitate knowledge graph-based fault prediction.

## Figures and Tables

**Table 1:** Data type.

Data Sources	Data Fields	Data Type
System Manual	Functional Description	Unstructured data
	Component Description	Semi-structured data
Typical Failure Cases	Fault Phenomenon	Unstructured data
	Cause of Failure	Unstructured data
	Troubleshooting Process	Unstructured data
	Fault Impact	Unstructured data

**Table 2:** Text Categories and Examples.

Text Category	Typical Example
Fault Phenomenon	Electro-hydraulic servo valve housing oil seepage
Troubleshooting Process	Replacement of machine parts
Cause of Fault	Electro-hydraulic servo valve housing not sealing
fault impact	Insufficient pressure in electro-hydraulic servo valve

**Table 3:** Training parameters for text classification models.

Parameters	Value
Batch size	8
Learning rate	0.00003
Epochs	10
Max length	128
Optimiser	Adam
Dropout	0.1

**Table 4:** Text Classification Test Results.

Model	*p*	*R*	*F*_1_
BERT	90.57	90.68	90.63 ± 0.01
BERT_CNN	90.61	90.51	90.56 ± 0.02
BERT_RNN	90.94	90.92	90.93 ± 0.00
BERT_RCNN	91.20	91.24	91.22 ± 0.00
BERT_DPCNN	90.39	90.35	90.37 ± 0.02
ERNIE	94.49	94.53	94.51 ± 0.02

**Table 5:** Training parameters for text classification models.

Parameters	Value
Batch size	8
Learning rate	0.00003
Epochs	10
Max length	128
Optimiser	Adam
Dropout	0.1

**Table 6:** Text Classification Test Results.

Model	*p*	*R*	*F*_1_
Casrel	69.492	74.738	72.114 ± 0.035
GPlinker	72.479	76.753	74.616 ± 0.033
BiLSTM-CRF-TreeBiLSTM	60.753	62.859	61.806 ± 0.022
ERNIE-BiLSTM-CRF-TreeBiLSTM*	76.524	79.848	78.186 ± 0.031
ERNIE-BiLSTM-CRF-TreeBiLSTM	83.441	86.737	85.089 ± 0.032

ERNIE-BiLSTM-CRF-TreeBiLSTM* for models without text classification.

**Table 7:** Development Environment and Version Information Sheet.

Development Environment	Tools and Version Information
CPU	Intel Core i7-10750H@2.60GHz
GPU	NVIDIA GeForce GTX 1660ti
Development tool	Visual Studio Code1.84.0
Databases	Neo4j 3.4.0
Dependent environment	Python 3.9.13, Vue 2.6.11

## Data Availability

Data is unavailable due to privacy.
